# Supplementary material for: ToxDAR: A Workflow Software for Analyzing Toxicologically Relevant Proteomic and Transcriptomic Data, from Data Preparation to Toxicological Mechanism Elucidation
Source: Int J Mol Sci. 2024 Sep 2;25(17):9544. doi: 10.3390/ijms25179544 (PMC11394870; doi:10.3390/ijms25179544)
Supplement: Supplementary file 1 [file ijms-25-09544-s001.zip › Supplementary Material S3 - Supplementary Methods.pdf]

Supplementary Methods

1. Ten normalization methods:

In our article, we integrated ten normalization methods: the median of the ratios of observed counts (DESeq), upper quartile (UQ), Trimmed Mean of M values (TMM), Total Ubiquitous (TU), Total Read Count (TC), Total Read Number (TN), External RNA Control Consortium (ERCC), Housekeeping Genes (HG7), Cellular RNA (CR), and Nuclear RNA (NR). These methods offer significant advantages in normalizing omics data.

(1) The DESeq method is a differential expression analysis approach based on a negative binomial distribution model. Its underlying principle involves linking variance to mean through local regression, allowing for precise estimation of gene expression variability. The research team led by Simon Anders tested DESeq across various RNA-Seq datasets and found that it not only employs a more robust size estimation formula but also offers faster computation and a more straightforward conceptual framework[1]. Juliana Costa-Silva's team evaluated several RNA-Seq differential expression analysis methods using qRT-PCR data and concluded that DESeq2 provides the most balanced performance in terms of precision, accuracy, and sensitivity[2].

(2) The Upper Quartile (UQ) method is a statistical approach designed to reduce the impact of extreme values on estimation accuracy. It works by normalizing the raw expression levels of each gene by dividing them by the upper quartile value. Pierre R. Bushel's team evaluated seven different normalization methods on human HepaRG cell control samples and found that the Upper Quartile (UQ) method performed best in maintaining fold-change (FC) levels[3]. Bullard et al. further validated that the UQ method's results were consistent with those obtained using edgeR, with agreement up to 10 decimal places, demonstrating that UQ is a rigorously tested and reliable tool suitable for high-precision data normalization[4].

(3) The Trimmed Mean of M-values (TMM) method is a statistical approach designed to address the impact of sequencing depth differences between samples on gene expression comparisons. The principle behind TMM involves calculating log-ratios, removing extreme values, and computing the mean to normalize gene expression data. Robinson and colleagues successfully applied the TMM method to normalize samples with varying types and amounts of RNA, effectively reducing the artifacts caused by differences in RNA composition between samples[5]. Yingdong Zhao and others found that, in downstream analyses of PDX RNA-seq data, the TMM method outperformed TPM and FPKM methods in cross-sample comparisons and differential expression analysis[6].

(4) The Total Ubiquitous (TU) method is a normalization technique used in gene expression data analysis. It operates by identifying genes that are ubiquitously expressed across most samples and uses the expression levels of these genes to calculate a global normalization factor between samples.

(5) The Total Read Count (TC) method is a simple and intuitive normalization approach where the raw read count for each gene is divided by the total read count (library size) of that sample, thereby reducing the impact of sequencing depth on gene expression levels.

(6) The Total Read Number (TN) method follows the same principle as the Total Read Count (TC) method. However, in certain contexts, TN may refer to the total number of reads, while TC could specifically refer to the reads used for analysis. In studies involving the NormExpression package, normalization using the TU, TC, and TN methods has been demonstrated to be effective in both

single-cell RNA-seq and bulk RNA-seq data[7].

(7) The External RNA Control Consortium (ERCC) method is an RNA sequencing normalization technique that uses external RNA control samples of known concentrations to correct for technical variations and differences in sequencing depth in gene expression data. Alison S. Devonshire and her team evaluated the utility of ERCC RNA standards in normalizing gene expression biomarker measurements. Their study found that ERCC RNA standards provide a universal approach to assess various aspects of platform performance and can offer technical variation information related to the quantification of biomarkers across different physiological abundance levels. The different combinations of these standards can serve as an ideal quality control toolkit for determining the accuracy of differences between normal and disease profiles[8].

(8) The Housekeeping Genes (HG7) method is a gene expression data normalization technique that uses a set of stably expressed reference genes to correct for differences in gene expression between samples. In experiments such as quantitative PCR (qPCR) or Northern blot, the relative expression changes of the target gene can be assessed by comparing its expression level to that of the Housekeeping Genes[9].

(9) The Cellular RNA (CR) method is a normalization technique that uses the total RNA content within cells to correct for technical variations and differences in sequencing depth in gene expression data. Deeptiman Chatterjee and his team discussed the standardization of single-cell RNA sequencing workflows, which is a crucial step in studying cell-type-specific gene expression in tissues such as Drosophila ovaries. The paper highlights various methods for normalizing single-cell RNA sequencing data, including the CR method, which contribute to improving the accuracy and reliability of data analysis[10].

(10) The Nuclear RNA (NR) method is a normalization technique that uses the amount of RNA within the cell nucleus to correct for technical variations and differences in sequencing depth in gene expression data. Ding et al. conducted a systematic comparison of single-cell and single-nucleus RNA sequencing methods. Their study provides an in-depth analysis of various scRNA-seq approaches, demonstrating the practical application and importance of using the NR method for normalization. The research highlights the advantages of the NR method in improving data quality and analysis accuracy. This approach enables researchers to gain a deeper understanding of gene expression regulation mechanisms and their roles in different cell types or disease states[11].

2. Functional enrichment analysis

We investigate the most commonly used method for differential gene enrichment analysis—the hypergeometric distribution[12], which has been applied in hundreds of published studies. This method consists of three steps: identifying differentially expressed genes, annotating these genes with respect to their involvement in pathways and processes, and performing statistical tests to determine whether these genes are significantly enriched in biological processes.

In the first step, differentially expressed genes are identified using statistical models, and genes with a p-value < 0.05 are selected as significantly differentially expressed. The probability of gene expression differences is also estimated. In the second step, annotation is performed through Gene Ontology (GO) terms[13] or Kyoto Encyclopedia of Genes and Genomes (KEGG) pathways[14] to determine the roles of these genes in biological processes. In the final step, the hypergeometric distribution is used for functional enrichment analysis of differential genes. The hypergeometric distribution describes the statistical probability of the number of genes in a specific function or pathway relative to the total number of genes in the differential expression gene set, helping us determine the extent of significant enrichment in specific functions or pathways. Genes with a p-value < 0.05 are selected as significantly differentially expressed gene sets. Since functional enrichment analysis involves multiple categories, p-values for each category need to be adjusted for multiple comparisons to control the false discovery rate.

In our functional enrichment analysis, the underlying functional data used are GOCC, GOBP, GOMF, and KEGG: (1) Cellular Component (CC): This level describes the localization of gene products (e.g., proteins) within the cell. It helps to understand the function and pathways of gene products within the cell. (2) Biological Process (BP): This level describes the biological processes that the genes participate in. It helps to understand the physiological functions of the organism and the mechanisms of disease development. (3) Molecular Function (MF): This level describes the molecular-level functions of gene products, which helps in understanding their interactions with other molecules or in catalyzing biochemical reactions. (4) KEGG enrichment analysis is a database resource used for functional annotation and pathway enrichment analysis of a set of genes. By comparing the gene set with pathway annotations in the KEGG database, it identifies pathways that are significantly enriched in specific biological processes or diseases. This helps provide a deeper understanding of the functional impact of the gene set.

Reference

1. Anders S, Huber W. Differential expression analysis for sequence count data, Genome Biology 2010.
2. Costa-Silva J, Domingues D, Lopes FM. RNA-Seq differential expression analysis: An extended review and a software tool, PLOS ONE 2017.
3. Bushel PR, Ferguson SS, Ramaiahgari SC et al. Comparison of Normalization Methods for Analysis of TempO-Seq Targeted RNA Sequencing Data, Frontiers in Genetics 2020.
4. Bullard JH, Purdom E, Hansen KD, Dudoit S. Evaluation of statistical methods for normalization and differential expression in mRNA-Seq experiments, BMC Bioinformatics 2010.
5. Robinson MD, Oshlack A. A scaling normalization method for differential expression analysis of RNA-seq data, Genome Biology 2010.
6. Zhao Y, Li M-C, Konaté MM et al. TPM, FPKM, or Normalized Counts? A Comparative Study of Quantification Measures for the Analysis of RNA-seq Data from the NCI Patient-Derived Models Repository, Journal of Translational Medicine 2021.
7. Wu Z, Liu W, Jin X et al. NormExpression: An R Package to Normalize Gene Expression Data Using Evaluated Methods, Frontiers in Genetics 2019.
8. Devonshire AS, Elaswarapu R, Foy CA. Evaluation of external RNA controls for the standardisation of gene expression biomarker measurements, BMC Genomics 2010.
9. Kouadjo KE, Nishida Y, Cadrin-Girard JF et al. Housekeeping and tissue-specific genes in mouse tissues, BMC Genomics 2007.
10. Chatterjee D, Deng W-M. Standardization of Single-Cell RNA-Sequencing Analysis Workflow to Study Drosophila Ovary, Methods in Molecular Biology 2023.
11. Ding J, Adiconis X, Simmons SK et al. Systematic comparison of single-cell and single-nucleus RNA-sequencing methods, Nature Biotechnology 2020.
12. Bleazard T, Lamb JA, Griffiths-Jones S. Bias in microRNA functional enrichment analysis, Bioinformatics 2015.
13. Ashburner M, Ball CA, Blake JA et al. Gene ontology: tool for the unification of biology. The Gene Ontology Consortium, Nature Genetics 2000.
14. Kanehisa M, Goto S. KEGG: kyoto encyclopedia of genes and genomes, Nucleic Acids Research 1999.
