# Supplementary material for: ToxDAR: A Workflow Software for Analyzing Toxicologically Relevant Proteomic and Transcriptomic Data, from Data Preparation to Toxicological Mechanism Elucidation
Source: Int J Mol Sci. 2024 Sep 2;25(17):9544. doi: 10.3390/ijms25179544 (PMC11394870; doi:10.3390/ijms25179544)
Supplement: Supplementary file 1 [file ijms-25-09544-s001.zip › Supplementary Material Table S1 - Classification System of Toxic Substances.pdf]

Classification System of Toxic Substances

Toxic classification methods	Representative toxic substances	Implication
1. To categorize toxins based on their mode of action, origin, and application	**1.1 Corrosive Toxic Substances**	Refers to poisons whose main toxic effect is local corrosion, such as strong acids, strong alkalis, phenols, silver nitrate, and copper salts.
	**1.2 Metallic toxins (parenchymal toxins)**	Refers to all metal poisons that damage the parenchymal cells of organ tissues and produce varying degrees of morphological changes, such as arsenic, mercury, lead, barium, and other heavy metal salts.
	**1.3 Cerebrospinal dysfunction poison**	Refers to poisons that, after entering the body, change the function of the brain and spinal cord and cause poisoning symptoms, such as barbiturates, phenothiazines, and other hypnotic sedatives, various anesthetics, stimulants, hallucinogens, alcohol, and most drugs.
	**1.4 Respiratory dysfunction poison**	Refers to poisons that, after entering the body, cause respiratory dysfunction leading to hypoxia and suffocation as the main characteristic, such as cyanide, carbon monoxide, hydrogen sulfide, and nitrite, etc.
	**1.5 pesticide**	Refers to agents mainly used to prevent and control pests and diseases that harm crops and agricultural products and to remove weeds, such as organophosphorus, organic nitrogen, carbamates, pyrethroids, etc., and also includes plant growth regulators.
	**1.6 Rodenticide**	Refers to poisons mainly used to kill rodents, such as zinc phosphide, Diphacinone-sodium, fluoroacetamide, tetramine, and other inorganic or organic synthetic rodenticides. Sometimes rodenticides are also included in the category of pesticides.
	**1.7 Poisonous animals**	Refers to animals whose organs and tissues are poisonous in whole or in part, such as venomous snakes, pufferfish, blister beetles, fish gallbladders.

	**1.8 Poisonous plants**	Refers to poisonous plants, such as Aconitum, Gelsemium elegans, Tripterygium wilfordii, and poisonous mushrooms.
	**1.9 Bacterial and fungal toxins**	Refers to toxins produced by pathogenic microorganisms, such as Pseudomonas cocovenenans toxin, botulinum toxin, and Fusarium toxin.
**2. Classify toxins based on their chemical properties**	**2.1 Volatile poisons**	Refers to poisons separated by distillation or micro-diffusion, such as alcohols, cyanides, phenols, chloral hydrate, organophosphorus, and organochlorine pesticides.
	**2.2 Non-volatile poisons**	Refers to poisons that cannot evaporate with water vapor but dissolve in organic solvents and are separated by organic solvent extraction. They are divided into three categories: acidic, alkaline, and amphoteric poisons. They include acidic poisons: barbiturates, cantharidin; alkaline poisons: alkaloids, phenothiazine sedatives, etc.; amphoteric poisons: morphine.
	**2.3 Metal poisons**	Refers to toxic substances separated by destroying organic matter, such as arsenic, mercury, barium, copper, and lead.
	**2.4 Anionic poisons**	Refers to poisons separated by dialysis or ion exchange, such as strong acids, strong bases, and nitrites.
	**2.5 Other poisons**	Refers to poisons that require special methods to separate and extract due to their chemical properties, such as carbon monoxide, curare, and hydrogen sulfide.
**3. To categorize toxins based on their toxicological effects**	**3.1 Corrosive poison**	Refers to a poison that has a strong corrosive effect on the area it contacts.
	**3.2 Substantial poison (called destructive poison)**	Refers to poisons that cause obvious pathological morphological damage to solid organs (liver, kidney, heart, brain.) after absorption, such as metal poisons, zinc phosphide, and certain poisonous mushrooms.
	**3.3 Enzyme system poisons**	Refers to poisons that mainly inhibit the activity of specific enzyme systems after being absorbed, such as organophosphorus pesticides, cyanide, carbon disulfide, and sodium pentachlorophenol.

	**3.4 Blood poison**	Refers to poisons that mainly cause changes in the blood after being absorbed, such as carbon monoxide, nitrite, hydrogen sulfide, nitrobenzene, certain snake venoms, etc.
	**3.5 Nerve poison**	Refers to poisons that mainly impair the function of the central nervous system after absorption, such as alcohol, barbiturates and non-barbiturate hypnotic sedatives, anesthetics, strychnine, nicotine, and cocaine.
**4. To categorize toxins based on their scope of application**	**4.1 Industrial poisons**	It includes raw materials, intermediates, auxiliary agents used in industrial production, as well as products, by-products, impurities or "three wastes" generated during the production process, such as strong acids, strong alkalis, solvents (such as gasoline, benzene, toluene, xylene), methanol, formaldehyde, phenol, and ethanol.
	**4.2 Agricultural poisons (pesticides)**	Agricultural poisons (pesticides) refer to agents that are mainly used to prevent and control pests and diseases that harm crops and agricultural products and to remove weeds, such as organophosphorus, organic nitrogen, carbamates, pyrethroids, etc., and also include plant growth regulators.
	**4.3 Environmental pollutants**	Including industrial and domestic waste gas, sewage, solid pollutants, and domestic garbage.
	**4.4 Food Chemicals and Food Toxins**	Including natural and synthetic preservatives, pigments and other food additives, natural toxins, and toxins produced when food spoils.
	**4.5 Daily necessities and hobbies**	Including cosmetics, hair dye, oil paint, mosquito coils, tobacco, and alcohol.
	**4.6 Medicines and medical supplies**	Refers to drugs originally used to prevent and treat diseases, which can become poisons due to overdose or improper use. It includes human and veterinary drugs, diagnostic and therapeutic supplies, such as reagent diagnostic kits and infusion equipment.
	**4.7 Biological toxins**	Including animal and plant toxins, such as snake venom, scorpion venom, insect venom, and bacterial toxins.

	**4.8 Military poison**	Refers to various toxic substances used as chemical weapons in war, mainly poison gas such as sarin and mustard gas.
	**4.9 Radioactive Elements**	That is, elements and rays with radioactive energy.
