# Supplementary material for: ToxDAR: A Workflow Software for Analyzing Toxicologically Relevant Proteomic and Transcriptomic Data, from Data Preparation to Toxicological Mechanism Elucidation
Source: Int J Mol Sci. 2024 Sep 2;25(17):9544. doi: 10.3390/ijms25179544 (PMC11394870; doi:10.3390/ijms25179544)
Supplement: Supplementary file 1 [file ijms-25-09544-s001.zip › Supplementary Material Table S2 - Representative Toxic Information Under Each Classification System.pdf]

Representative Toxins for Five Types

Toxin types	Representative Toxic Substances				
	Names of toxins	DrugBank IDs	Toxicological pathways	Toxicological molecules	References
Corrosive toxicants	Ammonia	DB11118	TLR4/TNF-α signaling pathway	muc2、claudin-1、IL-6、IL-10，TLR4、MyD88、NF-κB、TNF-α、IL-1β、caspase3	Ecotoxicol Environ Saf. 2021;226:112832.
			Wnt/β-catenin signaling pathway	RNF43	Environ Pollut. 2022;292(Pt A):118332.
			mTOR signaling pathway<br>AMPK signaling pathway	FASN、SCD、 FADS1、LDLR、PNPLA4, ANGPTL4、CEL、CPT1A, CPT1B、CPT2、ACADL、ACADVL、 UCP2、UCP3	Sci Total Environ. 2020;740:139917.
			Nrf2-Keap1 signaling pathway	SOD、CAT、GPX、Nrf2、Keap1	Aquat Toxicol. 2021;240:105969.
			NF- κB signaling pathway	Nrf2、Keap1、HSP70、HSP90、p50、p65、BAFF	Toxicol. 2020;229:105621.

			Glutamate and aspartate metabolic pathways		J Clin Invest. 1977 Mar;59(3):386-96.
			NAD+ metabolic pathways		J Neurochem. 2004 Jun;89(5):1101-10.
	Formaldehyde	DB03843	Akt/CREB signaling pathway	TNFAIP1	Neurotox Res. 2020;38(1):184-198.
			Nitric oxide-cGMP signaling pathway		Chemosphere. 2018;193:60-73.
			T-cell receptor signaling pathway		Sci Rep. 2022;12(1):8149.
**Substantial poison (destructive poison)**	arsenic		ROS signaling pathway	Bcl-xl、Bcl-2、bax、bak、keap1、MDM2、HIF-1、NF- κB、AP-1、eIF4E、eEF2、AP-1、RAS、STAT、c-src、HIF-1、P53	Ecotoxicol Environ Saf. 2021;208:111752.
			PI3K/AKT signaling pathway		
			mTOR signaling pathway		
			EGFR signaling pathway		
			NF- κB signaling pathway		
			Apoptosis signaling pathway		
	mercury		JNK/p38 MAPK signaling pathway	JNK、p38	J Trace Elem Med Biol. 2022;74:127057.

			Apoptosis pathway		
	lead		Nrf2/Keap1 signaling pathway	Nrf2、Keap1	Ecotoxicol Environ Saf. 2021;207:111231.
			Oxidative Phosphorylation Pathway		Kidney Int. 2003;63(1):186-194.
	thallium		ROS signaling pathway<br>Caspase signaling pathway	Bcl-xl、Bcl-2、Bad、Bax、PAF1	Adv Neurobiol. 2017;18:345-353.
	chromium	DB11136	ERK1/2 signaling pathway<br>p53 signaling pathway	PGC-1α、NRF-1、TFAM、p53	Chemosphere. 2021;271:129735.
			ROS-ATF6-PLK4 pathway	ATF6、PLK4	Cell Biol Int. 2022;46(7):1128-1136.
**Enzyme system poisons**	cyanides		HIF-1α/BNIP3 signaling pathway<br>cellular death pathways	BNIP3、p38、HIF-1α	Free Radic Biol Med. 2007;43(1):117-127.
	organophosphorus pesticide		cholinergic signaling pathway	p38、TNF-α、p21、SOCS3、STAT5、AP1、ELK1、NFAT	Int J Mol Sci. 2022;23(9):4523.
**Blood**	carbon monoxide	DB11588	ROS signaling pathway	NF- κB、TNF-α、Bcl-2、Bcl-X、	Am J Physiol Cell Physiol.

**toxins**			TLR4/ NF- κB signaling pathway	p38	2018;314(2):C211-C227.
			MKK3/p38 MAPK signaling pathway		
			Apoptosis pathway		
	nitrite	DB12529	Oxidative Phosphorylation Pathway		J Aquat Anim Health. 2022;34(2):58-68.
	hydrogen sulfide		CYP450s/ROS signaling pathway	TNF-α、NF- κB、Fas、FADD	Chemosphere. 2019;214:25-34.
			MAPK/ NF- κB signaling pathway	RIPK1、RIPK3、MLKL、TAK1、1AB2、TAB3、NF- κB、TNF-α	Oxid Med Cell Longev. 2019;2019:8061823.
			FOS/IL8 signaling pathway	FOS	J Hazard Mater. 2019;368:243-254.
			Energy Metabolic Pathways		Environ Res. 2018;167:1-6.
**Neurotoxic Substances**	Ethanol	DB00898	p53 signaling pathway	p53	Int J Mol Sci. 2021;22(18):9686.
			ROS signaling pathway		
	methanol		Apoptosis pathway		J Neurobiol. 2004 Sep 5;60(3):308-18.
	Chlorpromazine	DB00477	TIRAP signaling pathway	TIRAP、TNF-α	Toxicol Appl Pharmacol. 2013;266(3):430-438.

			Akt/mTOR pathway	mTOR、Akt	Carcinogenesis. 2013;34(9):2080-2089.
	nicotine	DB00184	mTOR signaling pathway<br>JAK-STAT3 signaling pathway<br>PI3K/AKT signaling pathway<br>P38/ERK/JNK signaling pathway<br>IκB/ NF- κB signaling pathway<br>Nrf2/HO-1 signaling pathway	mTOR、STAT3、Akt、P38、NF- κB、Nrf2	Front Immunol. 2022;13:826889.
	Phenobarbital	DB01174	Citric Acid Cycle Pathway		Anesthesiology. 1978 Mar;48(3):175-82.
			Glycolysis Pathway		
